# CMSP suppresses oral squamous cell carcinoma progression by targeting the JAK2/STAT3/c-Myc axis

**DOI:** 10.3389/fonc.2025.1671797

**Published:** 2025-11-19

**Authors:** Yueting Lu, Manman Yao, Dixian Wang, Daisuke Higuchi, Hualin Lu, Hongyue Shang, Bo Dong, Jiao Zhang, Ruizhe Jin, Tiejun Liu

**Affiliations:** 1 Department of Stomatology, The Fourth Hospital of Hebei Medical University, Shijiazhuang, China; 2 Department of Prosthodontics, Matsumoto Dental University, Matsumoto, Japan; 3 Department of Stomatology, Hebei Children’s Hospital, Shijiazhuang, Hebei, China

**Keywords:** oral squamous cell carcinoma, p-hydroxycinnamaldehyde, JAK2/STAT3/c-Myc signaling pathway, epithelial-mesenchymal transition, apoptosis, transcriptome sequencing, tumor immune microenvironment

## Abstract

**Background:**

Oral squamous cell carcinoma (OSCC) is a highly invasive head and neck malignancy with poor prognosis and limited treatment efficacy. This study aimed to investigate the anti-tumor potential of p-hydroxycinnamaldehyde (CMSP), a bioactive compound derived from the traditional Chinese and Mongolian medicinal herb Momordica cochinchinensis.

**Methods:**

The effects of CMSP on OSCC were evaluated in vitro using CAL27 and SCC15 cell lines and in vivo in a CAL27 xenograft nude mouse model. Cell proliferation, migration, and invasion were assessed by CCK-8 and transwell assays. Flow cytometry was used to analyze cell cycle and apoptosis. Transcriptomic sequencing followed by KEGG and GO enrichment analyses was performed to identify key regulatory pathways, and Western blotting was used to validate protein expression. Bioinformatics and molecular docking analyses were further conducted to explore CMSP–target interactions.

**Results:**

CMSP inhibited proliferation, migration, and invasion of OSCC cells in a dose-dependent manner, induced S-phase arrest, and promoted apoptosis. Transcriptomic and enrichment analyses identified the JAK2/STAT3 signaling pathway as a major target. Western blotting confirmed that CMSP significantly suppressed phosphorylation of JAK2 and STAT3 and downregulated downstream c-Myc expression. In vivo, CMSP markedly reduced tumor growth in nude mice. Bioinformatics and molecular docking suggested that MYC-related signaling contributes to the anti-tumor activity of CMSP in OSCC.

**Conclusion:**

CMSP exerts anti-OSCC effects, at least in part, through modulation of the JAK2/STAT3/c-Myc signaling axis, and may serve as a promising adjunctive therapeutic candidate for OSCC management.

## 1 Introduction

Oral squamous cell carcinoma (OSCC) is the most common malignancy of the head and neck, with approximately 350,000 new cases worldwide each year and a 5-year survival rate of around 50% (1, 2). Although surgical resection, radiotherapy, and chemotherapy have improved, most patients still face poor outcomes due to the aggressive nature, metastatic potential, and therapeutic resistance of OSCC (3, 4). This highlights the need for new treatment strategies. In recent years, extracts from traditional Chinese medicine (TCM) have gained attention in anti-tumor drug development. Many such extracts demonstrate multi-target anti-cancer effects. Notably, Tu Youyou was awarded the Nobel Prize for the discovery of artemisinin from Artemisia annua. Currently, more than 200 TCM extracts are used in cancer therapy, either alone or in combination with conventional treatments, and have shown benefits such as enhanced efficacy, reduced toxicity, and fewer side effects (5, 6).

Momordicae Semen (Momordica seed) refers to the dried mature seeds of Momordica cochinchinensis Spreng, a plant in the Cucurbitaceae family. It has been widely used in TCM for its properties of resolving swelling and masses, dispelling toxins, and treating abscesses (7–9). Studies have demonstrated that various extracts of Momordicae Semen possess anti-cancer, anti-inflammatory, anti-ulcer, and immunomodulatory activities, and are often used in combination with other agents for cancer therapy (10–12). For instance, Shen et al. (13) reported that extracts from Momordicae Semen induced apoptosis in non-small cell lung cancer cells in a dose-dependent manner and inhibited metastasis by suppressing the *PI3K/AKT* pathway, regulating *PARP* protein expression, or activating the p53 pathway. In addition, Momordica saponins were found to increase the proportion of breast cancer cells in the G2 phase and the rate of apoptosis, thereby inhibiting cell proliferation (14).

Furthermore, p-hydroxycinnamaldehyde, a bioactive constituent isolated from the ethanol extract of Momordicae Semen, has been reported to exert potent anti-tumor effects. For clarity, we refer to this compound as CMSP (Cochinchina Momordica Seed Phytochemical) throughout this study.Zhao et al. found that CMSP inhibited malignant behaviors in esophageal squamous cell carcinoma and melanoma cells (7, 10, 15). Wang et al. showed that CMSP modulated the immune microenvironment and induced cell polarization by altering the proteome, thereby suppressing tumor growth (16). However, the effects of CMSP on OSCC cells remain unclear.

It is well established that the *JAK2/STAT3* signaling pathway is closely associated with the development and progression of OSCC. This pathway can promote EMT, immune evasion, and therapeutic resistance in OSCC through mediators such as chemerin and *interleukin-6 (IL-6)* (3, 17, 18). Additional evidence indicates that the *JAK2/STAT3/c-Myc* signaling axis plays a critical role in the pathogenesis and progression of OSCC (19, 20).

In this study, we systematically evaluated the anti-tumor activity of CMSP against OSCC. Various *in vitro* cellular assays were performed to investigate the effects of CMSP on cell functions. Transcriptomic sequencing and bioinformatics analyses were conducted to assess the regulatory impact of CMSP on the *JAK2/STAT3/c-Myc* pathway. In addition, an *in vivo* mouse xenograft model was employed to confirm the anti-tumor efficacy of CMSP. Collectively, this study elucidates the mechanism by which CMSP suppresses OSCC progression and provides a theoretical basis for the potential application of CMSP as a novel TCM-derived candidate for precision therapy of OSCC.

## 2 Materials and methods

### 2.1 Acquisition and storage of CMSP

CMSP(purity >98%) was purchased from Wuxi Flugu Pharmaceutical Technology Co., Ltd. (Jiangsu Province, China). CMSP was initially dissolved in dimethyl sulfoxide (DMSO) to prepare a stock solution at a concentration of 100 mg/ml. The stock solution was stored at –20°C until further use. For subsequent experiments, the stock solution was diluted with serum-free medium to the desired working concentrations.

### 2.2 Cell culture

The human OSCC cell lines CAL27 and SCC15 were obtained from Wuhan Procell Life Science & Technology Co., Ltd. (Wuhan, China) and Shanghai Zhong Qiao Xin Zhou Biotechnology Co., Ltd. (Shanghai, China), respectively. Both cell lines were cultured in Dulbecco’s Modified Eagle Medium (DMEM; Gibco, USA) supplemented with 10% fetal bovine serum (FBS; Gibco, USA) and 1% penicillin-streptomycin. Cells were maintained at 37°C in a humidified incubator with 5% CO_2_. Cells in the logarithmic growth phase were used for subsequent experiments.

### 2.3 Cell viability assay

The effect of CMSP on the viability of OSCC cells was evaluated using the MTS assay. OSCC cells in the logarithmic growth phase were seeded into 96-well plates at a density of 5 × 10³ cells per well in 200 μl of DMEM. After cell attachment, the medium was replaced with DMEM containing 0.01% DMSO (vehicle control) or varying concentrations of CMSP (0, 2, 4, 8, 10, or 20 μg/mL). Cells were incubated at 37 °C with 5% CO_2_ for 24, 48, or 72 hours. Following incubation, the medium was removed, and wells were gently washed with PBS. Subsequently, MTS reagent (Promega, USA) was added to each well and incubated in the dark according to the manufacturer’s instructions. Absorbance was measured at 490 nm using a microplate reader. Cell viability was calculated relative to the control group based on the standard curve.

### 2.4 Colony formation assay

The colony formation assay was performed to further evaluate the effect of CMSP on OSCC cell proliferation. CAL27 and SCC15 cells in the logarithmic growth phase were seeded into 6-well plates at a density of 8 × 10³ cells per well. After cell attachment, the medium was replaced with DMEM containing 0.01% DMSO (vehicle control) or CMSP at concentrations of 6, 8, or 10 μg/mL.Cells were incubated at 37°C with 5% CO_2_ for 7–10 days, with medium changes every 2–3 days as necessary. At the end of the incubation, the medium was removed, and colonies were washed twice with PBS, fixed with 4% paraformaldehyde for 15 minutes, and stained with 1% crystal violet for 20 minutes. After washing off excess dye, the number of colonies containing more than 50 cells was counted using ImageJ software.

### 2.5 Scratch (wound healing) and transwell invasion assays

#### 2.5.1 Wound healing assay

CAL27 and SCC15 cells in the logarithmic growth phase were seeded into 6-well plates at a density of 8 × 10^5^ cells per well. Upon reaching approximately 90% confluence, a linear scratch was made across the cell monolayer using a sterile 200 μl pipette tip. Detached cells were removed by washing twice with PBS. After aspirating residual liquid, 2 ml of serum-free DMEM containing 0.01% DMSO (vehicle control) or CMSP (2 or 4 μg/mL) was added to each well. Images of the wounded area were captured at 0 and 24 hours using an inverted microscope at the same location. The wound closure area was measured and quantified using ImageJ software.

#### 2.5.2 Transwell invasion assay

Cell invasion was evaluated using 24-well Transwell chambers (Corning, USA) pre-coated with Matrigel (BD Biosciences, USA) and allowed to solidify at room temperature. Cell suspensions (1 × 10^5^ cells in 100 μl serum-free DMEM) were seeded into the upper chamber. The lower chamber was filled with 500 μl of DMEM containing 10% fetal bovine serum as a chemoattractant, along with 0.01% DMSO or CMSP (2 or 4 μg/mL). After 24 hours of incubation at 37°C, non-invading cells on the upper surface of the membrane were removed with a cotton swab, and cells on the lower surface were fixed with 4% paraformaldehyde for 15 minutes, then stained with 1% crystal violet for 20 minutes. The number of invaded cells was counted in five randomly selected fields under a microscope and analyzed using ImageJ software.

### 2.6 Cell apoptosis and cell cycle analysis

CAL27 and SCC15 cells in the logarithmic growth phase were seeded into 6-well plates at a density of 3 × 10^5^ cells/well. After 6 h of attachment, cells were treated with CMSP (6, 8, or 10 μg/mL) or 0.01% DMSO (vehicle control) in complete DMEM for 48 h.

For apoptosis analysis, both adherent and floating cells were collected, washed twice with cold PBS, and resuspended in 100 μL of binding buffer. Cells were stained with 5 μL Annexin V-PE and 5 μL 7-AAD (BD Biosciences, USA) in the dark at room temperature for 15 min. Apoptotic cell populations were quantified as the percentage of Annexin V-positive cells, including both early (Annexin V^+^/7-AAD^-^) and late (Annexin V^+^/7-AAD^+^) apoptotic cells.Subsequently, 400 μL of binding buffer was added to each tube, and samples were analyzed using a FC500 flow cytometer (Beckman Coulter, USA). Data were analyzed using EXPO32 ADC 1.2 software.

For cell cycle analysis, similarly treated cells were fixed in 75% ethanol at 4 °C overnight. After washing twice with PBS, cells were stained with 500 μL of PI/RNase staining solution (BD Biosciences, USA) for 15 min in the dark. DNA content was measured using the same flow cytometer, and the distribution of cells in G0/G1, S, and G2/M phases was determined using EXPO32 ADC software (version 1.2, Beckman Coulter).

### 2.7 Transcriptome analysis

Total RNA was extracted from OSCC cells treated with 0.01% DMSO (control) or 8 μg/mL CMSP using Trizol reagent (Invitrogen, USA) according to the manufacturer’s protocol. RNA was further purified using the RNeasy Mini Kit (Qiagen, Germany). RNA concentration and purity were assessed using a Qubit^®^ 3.0 Fluorometer (Thermo Fisher Scientific, USA) and a NanoDrop One spectrophotometer (Thermo Fisher Scientific, USA). RNA integrity was evaluated by the Agilent 2100 Bioanalyzer (Agilent Technologies, USA), and only samples with an RNA integrity number (RIN) > 7.0 were used for subsequent library construction.

Paired-end mRNA sequencing libraries were prepared using the Illumina mRNA-Seq Library Prep Kit (Illumina, USA) following the manufacturer’s instructions. Sequencing was performed on an Illumina platform. Raw sequencing reads (fastq files) were aligned to the reference genome using HISAT2 (version 2.0.5). The resulting SAM (Sequence Alignment/Map) files were converted to sorted BAM (Binary Alignment/Map) files using SAMtools (version 1.3.1). Gene expression levels were quantified as fragments per kilobase of exon per million mapped reads (FPKM) using StringTie, and normalization was performed using the trimmed mean of M-values (TMM) algorithm.

Differential expression analysis was conducted using the edgeR package (version 3.4.3) in R. Genes with |log_2_ fold change| > 1 and p-value < 0.05 were considered significantly differentially expressed. Functional enrichment analyses, including Gene Ontology (GO; biological process, cellular component, molecular function) and Kyoto Encyclopedia of Genes and Genomes (KEGG) pathway analyses, were performed using the clusterProfiler package in R.

### 2.8 Quantitative real-time PCR analysis

RT-qPCR was performed to validate the transcriptome sequencing results. Total RNA was extracted from cells under the same experimental conditions described above. After measuring RNA concentration and purity, cDNA was synthesized using the RevertAid™ First Strand cDNA Synthesis Kit (Thermo Fisher Scientific, USA) according to the manufacturer’s protocol.

qPCR was conducted using GoTaq^®^ qPCR Master Mix (Thermo Fisher Scientific, USA) on an ABI Prism 7500 system (Applied Biosystems, USA). Gene-specific primers for SOCS2, MYC, DHFR, ARPC1B, ARL2BP, PCDHGC3, HMGB2, and the internal reference gene GAPDH were designed using Primer5 software. The primer sequences are as follows GAPDH (forward: 5’-TGTGGGCATCAATGGATTTGG-3’; reverse: 5’-ACACCATGTATTCCGGGTCAAT-3’),

SOCS2 (forward: 5’-CAGATGTGCAAGGATAAGCGG-3’; reverse: 5’-GCGGTTTGGTCAGATAAAGGTG-3’),

MYC (forward: 5’-GGCTCCTGGCAAAAGGTCA-3’; reverse: 5’-CTGCGTAGTTGTGCTGATGT-3’),

DHFR (forward: 5’-AGGCTAAGGCAGGCAGATCAC-3’; reverse: 5’-GCAGTGGCACAATCACGACTC-3’),

ARPC1B (forward: 5’-CAAGGACCGCACCCAGATT-3’; reverse: 5’-TGCCGCAGGTCACAATACG-3’),

ARL2BP (forward: 5’-TATCATGGATGACGAGTTCCAGT-3’; reverse: 5’-GGTGCTGTAATGTTGTGGTGAA-3’),

HMGB2 (forward: 5’-CCGGACTCTTCCGTCAATTTC-3’; reverse: 5’-GTCATAGCGAGCTTTGTCACT-3’).

Each reaction was performed in triplicate. GAPDH served as the endogenous control. The relative expression levels of target genes were calculated using the 2^−ΔΔCt method, where ΔCt = Ct(target gene) − Ct(GAPDH), and ΔΔCt = ΔCt(experimental group) − ΔCt(control group).

### 2.9 Construction of protein–protein interaction network

Down-regulated differentially expressed genes (DEGs) identified by transcriptome sequencing were imported into the STRING database (https://string-db.org/) to construct the (PPI). The minimum required interaction score was set to >0.4 (medium confidence), and the interaction results were exported in TSV format. The resulting PPI network was further visualized and analyzed using Cytoscape software (version 3.7.1), and key hub genes were identified based on the degree of connectivity.

### 2.10 Immune infiltration and survival analysis

The correlation between MYC expression and various immune-infiltrating cells in head and neck cancer, including OSCC, was analyzed using the TIMER database (https://cistrome.shinyapps.io/timer/).

Kaplan–Meier (K–M) survival analysis was performed to assess the prognostic impact of MYC expression on overall survival in OSCC patients. Clinical and gene expression data for OSCC were obtained from the GEO (https://www.ncbi.nlm.nih.gov/geo/), EGA (https://ega-archive.org/), and TCGA (https://portal.gdc.cancer.gov/) databases. Specifically, for the TCGA analysis, only samples from the HNSC cohort with primary tumor sites annotated as oral cavity subsites (e.g., tongue, buccal mucosa, floor of mouth, hard palate, alveolar ridge, and oral cavity NOS) were included. Cases originating from non-oral anatomical locations were excluded to ensure analysis specificity.

Survival curves were generated based on MYC expression levels, and statistical significance was assessed using the log-rank test, with a p-value < 0.05 considered statistically significant.

### 2.11 Molecular docking

Molecular docking was performed using AutoDock Vina (version 1.2.0) to predict the binding affinity between CMSP and target proteins relevant to OSCC. First, the three-dimensional structures of target proteins were downloaded from the Protein Data Bank (PDB; https://www.rcsb.org/). Non-protein molecules and water molecules were removed from the protein structures using PyMOL software (version 2.5.0), and the processed structures were saved in PDBQT format for docking. The 3D structure of CMSP was converted to PDB format using OpenBabel (version 3.1.1), then subjected to hydrogenation and energy minimization, and saved as PDBQT format.

### 2.12 Western blot analysis

After treatment, CAL27 and SCC15 cells were lysed using RIPA lysis buffer (Solarbio, China) supplemented with protease and phosphatase inhibitors. The total protein concentration was determined by the BCA assay. Equal amounts of protein samples were separated by sodium dodecyl sulfate–polyacrylamide gel electrophoresis (SDS–PAGE) and transferred onto polyvinylidene fluoride (PVDF) membranes (Millipore, USA) by electroblotting.

Membranes were blocked in TBST (Tris-buffered saline with 0.1% Tween-20) at room temperature for 30 minutes, with the buffer replaced every 10 minutes. The membranes were then incubated overnight at 4 °C with primary antibodies against *Vimentin(Proteintech Cat# 10366-1-AP, RRID: AB_2273020), E-cadherin(Proteintech Cat# 20874-1-AP, RRID: AB_10697811),caspase-3(Abmart Cat# T40044, RRID: AB_2936280), cleaved-caspase-3(WanLeiBio Cat# WL02117, RRID: AB_2910623), Bax(Proteintech Cat# 50599-2-Ig, RRID: AB_2061561),Bcl-2(Proteintech Cat# 127891AP,RRID: AB_2227948),CyclinA(WanLeiBioCat#WL01841,RRID: AB_3669086),CDK2(WanLeiBio Cat# WL01543, RRID: AB_3698084), c-Myc(Proteintech Cat# 10828-1AP,RRID: AB_2148585), JAK2(WanLeiBio Cat# WL02188, RRID: AB_3675337), phospho-JAK2(WanLeiBio Cat# WL02997, RRID: AB_3697825), STAT3(Affinity Biosciences Cat# AF6294, RRID: AB_2835144), phospho-STAT3(Affinity Biosciences Cat# AF3293, RRID: AB_2810278)*, and *β-actin(ServiceBio Cat# GB15003, RRID: AB_3083699)*, at appropriate dilutions according to the manufacturer’s instructions.

After washing, membranes were incubated with the appropriate secondary antibodies in the dark for 1 hour at room temperature. Protein bands were visualized using the Odyssey infrared imaging system (LI-COR, USA). The band intensity was quantified using ImageJ software, and the relative expression levels of target proteins were normalized to β-actin.

### 2.13 *In vivo* tumor xenograft assay

BALB/c nude mice (female, 4–6 weeks old, n = 10) were maintained under specific pathogen-free (SPF) conditions for one week prior to experimentation. To establish a tumor xenograft model, CAL27 cells (5 × 10^6^ cells/mouse) were suspended in PBS and subcutaneously injected into the right axilla of each mouse. Tumor formation was monitored daily, and five days after injection, mice with successfully established OSCC xenografts were randomly divided into two groups (n = 5 per group).

The treatment group received intraperitoneal injections of CMSP (20 mg/kg) every two days, starting on day 7 post-inoculation, while the control group received injections of 0.9% NaCl (normal saline) according to the same schedule. Tumor volumes were measured every two days using calipers and calculated as (length × width²)/2. After 19 days of treatment, all mice were sacrificed by cervical dislocation. Tumors were excised, washed with PBS, and weighed.

All animal procedures were performed in accordance with the principles of Replacement, Reduction, and Refinement (the 3Rs), and complied with relevant national regulations on animal welfare and ethics. The study protocol was approved by the Experimental Animal Ethics and Welfare Committee of the Fourth Hospital of Hebei Medical University (Approval No: IACUC-4th Hos Hebmu-20240601).

### 2.14 Statistical analysis

All statistical analyses were performed using GraphPad Prism version 8.3.0 (GraphPad Software, USA) and SPSS version 21.0 (IBM, USA). Data are presented as mean ± standard deviation (SD) from at least three independent experiments. Comparisons between two groups were conducted using the independent samples t-test. For comparisons among multiple groups, one-way analysis of variance (ANOVA) followed by Tukey’s *post hoc* test was used. A p-value < 0.05 was considered statistically significant.

## 3 Result

### 3.1 CMSP inhibits proliferation of OSCC cells

**Figure 1:**
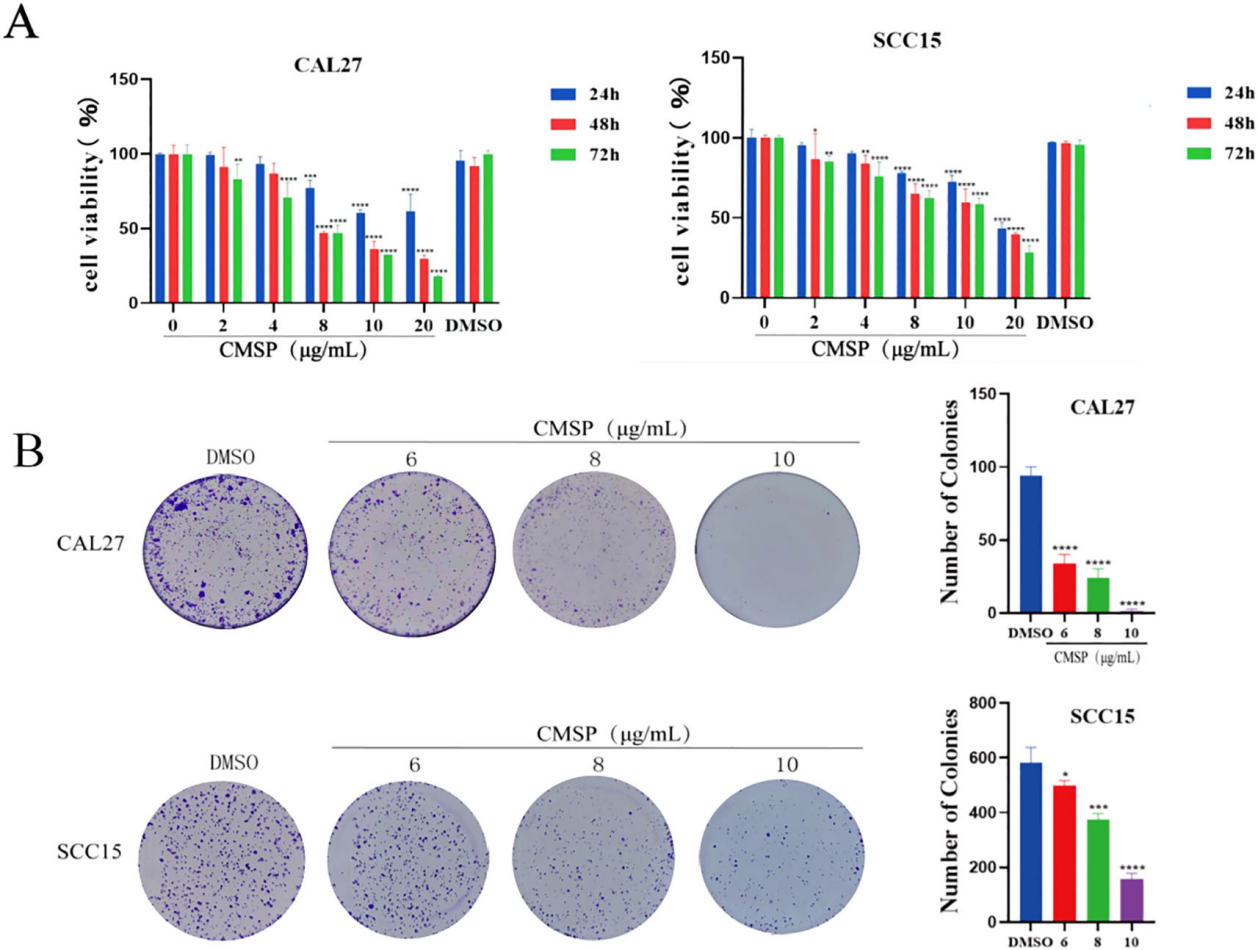
CMSP inhibits the proliferation of OSCC cells. **(A)** The absorbance of CAL27 and SCC15 cells was detected by MTS after 24, 48 and 72h treatment by CMSP. **(B)** Effects of different concentrations of CMSP (6, 8,10 μg/mL)on the cloning ability of CAL27 and SCC15 cells. n=3, compared with the control group, *P< 0.05, **P < 0.01, ***P < 0.001, ****P < 0.0001.

To evaluate the effect of CMSP on the proliferation of human OSCC cells, MTS assays were conducted using CAL27 and SCC15 cell lines. As shown in **Figure 1A**, CMSP treatment significantly suppressed cell viability in a time- and dose-dependent manner compared with the blank and solvent control groups at 24, 48, and 72 hours. The half-maximal inhibitory concentration (IC_50_) values of CMSP at 48 hours were calculated as 8.52 μg/mL for CAL27 cells and 13.84 μg/mL for SCC15 cells, indicating moderate cytotoxic activity.

In CAL27 cells, CMSP showed clear dose-dependent inhibition across the selected concentration range, which included both subcytotoxic and near-IC_50_ levels. In contrast, in SCC15 cells the applied doses were largely subcytotoxic; although 10 μg/mL induced significant inhibition, it may not fully reflect the cytotoxic potential of CMSP in this line.

To further validate the anti-proliferative effects of CMSP, colony formation assays were performed. Following treatment with CMSP at concentrations of 6, 8, and 10 μg/mL, both CAL27 and SCC15 cells exhibited a marked reduction in colony-forming ability in a dose-dependent manner, as illustrated in **Figure 1B**. These results corroborated the MTS data, collectively demonstrating that CMSP effectively inhibits the proliferative capacity of OSCC cells *in vitro*.

### 3.2 CMSP inhibits epithelial–mesenchymal transition in OSCC cells

**Figure 2:**
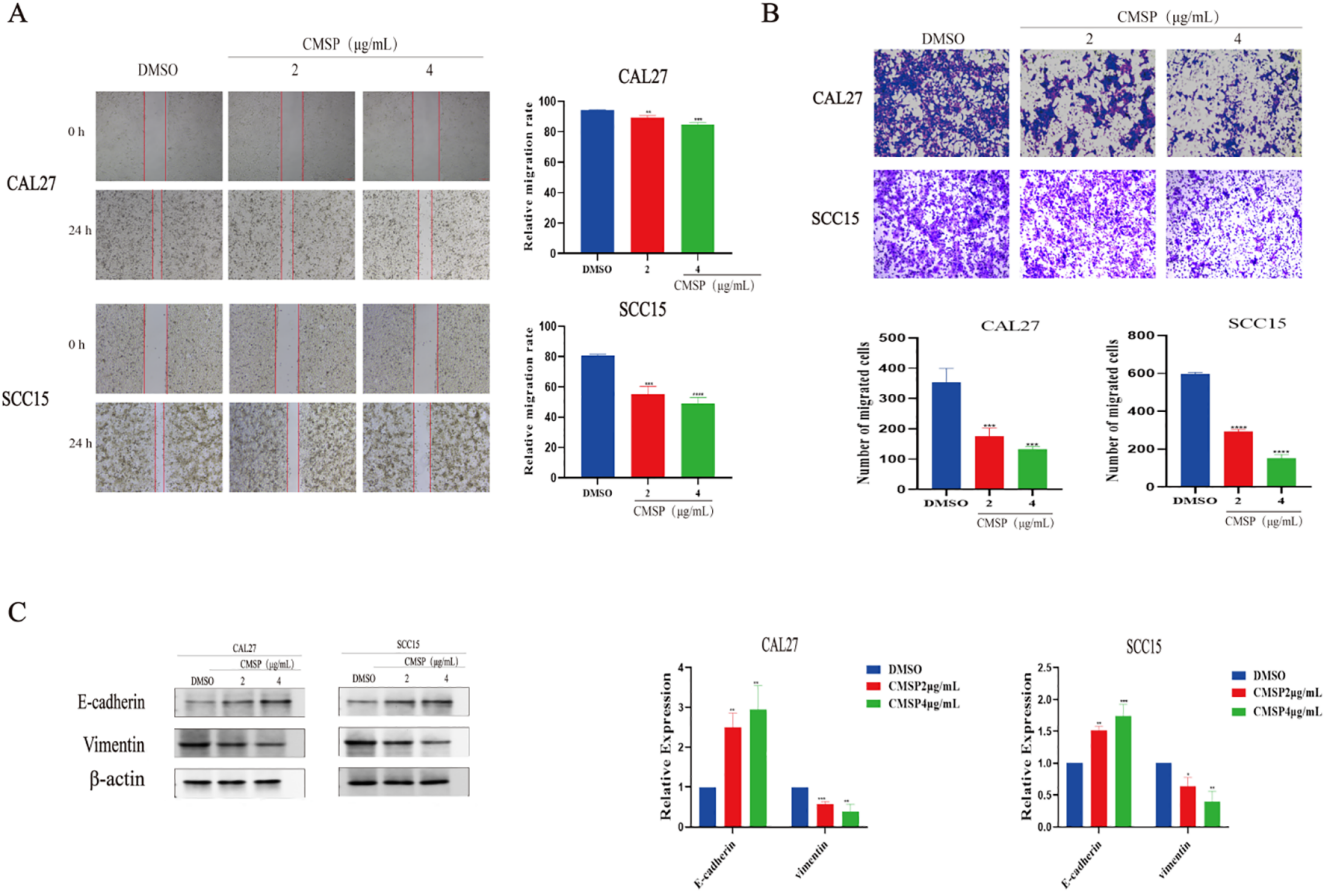
CMSP impairs the migration and invasion of OSCC cells. **(A)** Effects of different concentrations of CMSP (2, 4μg/mL) on migration ability of CAL27 and SCC15 cells treated for 24h. **(B)** Effects of different concentrations of CMSP (2, 4μg/mL) on invasion ability of CAL27 and SCC15 cells treated for 24h. **(C)** Effect of different concentrations of CMSP (2, 4μg/mL) on EMT-related protein expression in CAL27 and SCC15 cells treated for 24h. β-Actin to control the load. n=3, compared with the control group, *P< 0.05, **P < 0.01, ***P < 0.001, ****P < 0.0001.

To minimize the potential confounding effects of cytotoxicity on migration and invasion assessments, CAL27 and SCC15 cells were treated with relatively low concentrations of CMSP (2 and 4 μg/mL) for 24 hours. Cell migration ability was first evaluated using a wound healing assay. Compared with the solvent control group, CMSP significantly reduced the wound closure rate in both CAL27 and SCC15 cells, with a more pronounced effect observed at 4 μg/mL (**Figure 2A**). These findings indicate that CMSP effectively suppresses OSCC cell migration in a dose-dependent manner.

To further investigate its impact on invasive capacity, a Matrigel-coated transwell assay was performed. After 24 hours of treatment, the number of cells penetrating the matrix-coated membrane was significantly reduced in both cell lines exposed to CMSP, particularly at 4 μg/mL (**Figure 2B**). These results suggest that CMSP markedly impairs the invasive potential of OSCC cells.

Given that EMT is a fundamental mechanism underpinning tumor cell migration and invasion, we next examined the expression of EMT-related markers. Western blot analysis demonstrated a dose-dependent decrease in vimentin levels and a concomitant increase in E-cadherin expression following CMSP treatment in both cell lines (**Figure 2C**). These molecular alterations are consistent with the suppression of EMT.

Taken together, the data indicate that CMSP inhibits the migratory and invasive behavior of OSCC cells, at least in part, by interfering with the EMT process. This highlights a potential mechanism by which CMSP may exert its anti-metastatic effects in OSCC.

### 3.3 CMSP induces apoptosis in OSCC cells

**Figure 3:**
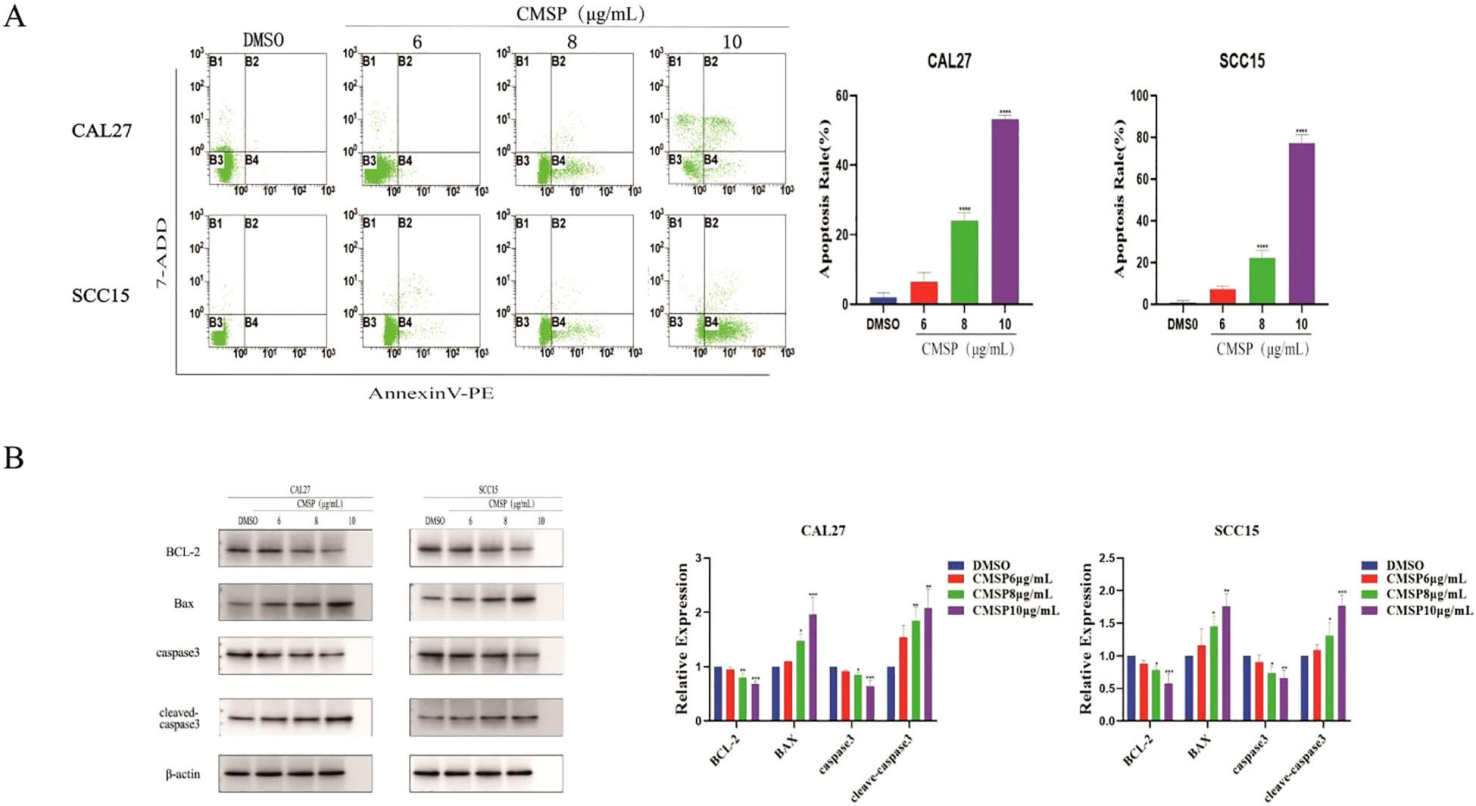
CMSP modulates apoptosis in OSCC cells. **(A)** Effects of different concentrations of CMSP (6, 8, 10μg/mL) on apoptosis rate of CAL27 and SCC15cells.The four quadrants represent: Q1 (Annexin V^-^/7-AAD^+^, necrotic cells), Q2 (Annexin V^+^/7-AAD^+^, late apoptotic cells), Q3 (Annexin V^-^/7-AAD^-^, viable cells), and Q4 (Annexin V^+^/7-AAD^-^, early apoptotic cells).The quantitative results show that CMSP treatment significantly increased the percentage of apoptotic cells in a dose dependent manner, with the highest apoptosis rate observed at 10 μg/mL in both cell lines. Data are presented as mean ± SD (n = 3). ****P < 0.0001 vs DMSO group. **(B)** Effects of different concentrations of CMSP (6, 8, and 10 μg/mL) on the expression of apoptosis-related proteins in CAL27 and SCC15 cells after 48 h of treatment.Protein expression levels of BCL-2, Bax, caspase-3, and cleaved caspase-3 were analyzed by Western blot. β-Actin was used as a loading control. Quantitative analysis of band intensity was performed using densitometry and is shown in the bar graphs.Data are presented as mean ± SD (n = 3). Compared with the control group: *P < 0.05, **P < 0.01, ***P < 0.001, ****P < 0.0001.

Apoptosis plays a pivotal role in inhibiting tumor cell proliferation and is a key target of many anti-cancer agents. To evaluate whether CMSP induces apoptosis in oral squamous cell carcinoma (OSCC) cells, CAL27 and SCC15 cells were treated with CMSP at concentrations of 6, 8, and 10 μg/mL for 48 hours. Flow cytometry using Annexin V-PE/7-AAD double staining demonstrated a significant, dose-dependent increase in apoptotic cell populations(Early apoptotic cells Annexin V^+^/7-AAD^-^, and Late apoptotic cells Annexin V^+^/7-AAD^+^). At 10 μg/mL CMSP, the apoptotic rate reached 52.26% in CAL27 and 73.16% in SCC15 cells, markedly higher than the DMSO control group (**Figure 3A**, P < 0.0001).

To further elucidate the mechanism underlying CMSP-induced apoptosis, Western blot analysis was performed to assess the expression of key apoptotic regulatory proteins. As shown in **Figure 3B**, CMSP treatment (8 and 10 μg/mL, 48 h) significantly increased the levels of cleaved caspase-3 and the pro-apoptotic protein Bax, while reducing the expression of the anti-apoptotic protein BCL-2. The expression of total caspase-3 remained largely unchanged. These findings suggest that CMSP promotes apoptosis primarily through the activation of the intrinsic mitochondrial pathway.

Taken together, these results indicate that CMSP exerts its anti-proliferative effects in OSCC cells by triggering mitochondrial-mediated apoptosis via modulation of BCL-2 family proteins and activation of caspase signaling.

### 3.4 CMSP induces s phase cell cycle arrest in OSCC cells

**Figure 4:**
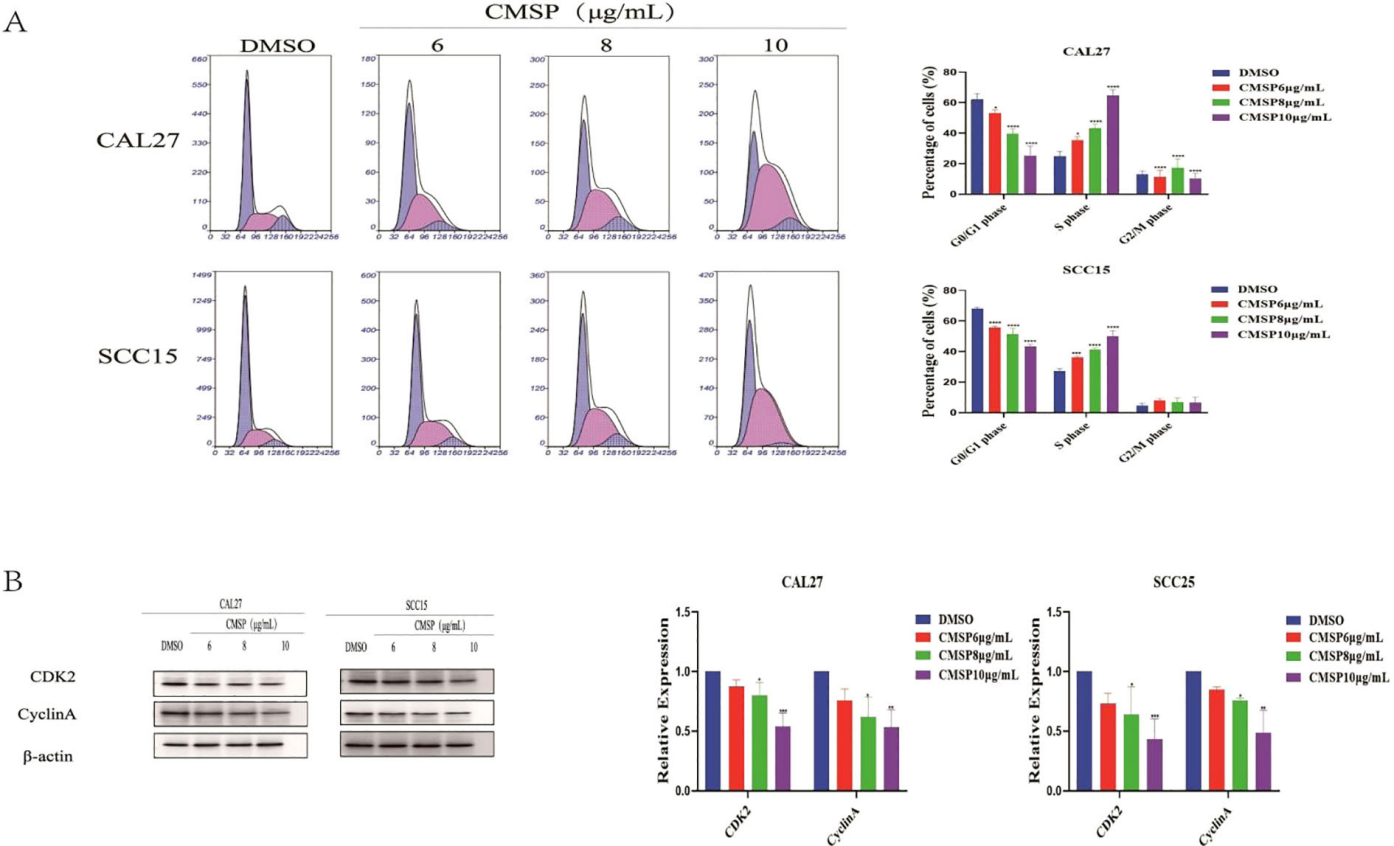
CMSP induces cell cycle arrest in OSCC cells. **(A)** CAL27 and SCC15 cells were treated with different concentrations of CMSP (6, 8, and 10 μg/mL) or 0.01% DMSO (control) for 48 hours. Cell cycle distribution was analyzed by flow cytometry following PI staining. Representative histograms and quantification of the percentage of cells in G0/G1, S, and G2/M phases are shown. **(B)** Western blot analysis of S-phase regulatory proteins CDK2 and Cyclin A in CAL27 and SCC15 cells after treatment with CMSP for 48 hours. β-Actin was used as the loading control. Bar graphs show relative protein expression levels normalized to β-Actin. Data represent mean ± SD from three independent experiments (n = 3). Statistical significance was assessed relative to the control group: *P < 0.05, **P < 0.01, ***P < 0.001, ****P < 0.0001.

Cell proliferation is tightly controlled by the cell cycle, and disruption of this process is a common mechanism by which anti-cancer agents exert their effects. To explore whether CMSP affects cell cycle progression in OSCC cells, flow cytometry was performed following treatment of CAL27 and SCC15 cells with CMSP at concentrations of 6, 8, and 10 μg/mL for 48 hours. The results demonstrated a significant, dose-dependent increase in the proportion of cells in the S phase, accompanied by a concomitant decrease in the G0/G1 phase population compared to the solvent control group (**Figure 4A**). These findings indicate that CMSP induces cell cycle arrest at the S phase.

To further elucidate the molecular mechanism underlying this arrest, Western blot analysis was conducted to assess the expression of S phase regulatory proteins CDK2 and Cyclin A. After 48-hour treatment with CMSP (8 and 10 μg/mL), both CAL27 and SCC15 cells exhibited a marked downregulation of CDK2 and Cyclin A protein levels in a dose-dependent manner (**Figure 4B**).

Together, these results suggest that CMSP interferes with OSCC cell cycle progression by suppressing key S phase-related proteins, thereby contributing to its anti-proliferative activity.

### 3.5 CMSP inhibits OSCC cell proliferation via suppression of the JAK2/STAT3/c-Myc signaling pathway

**Figure 5:**
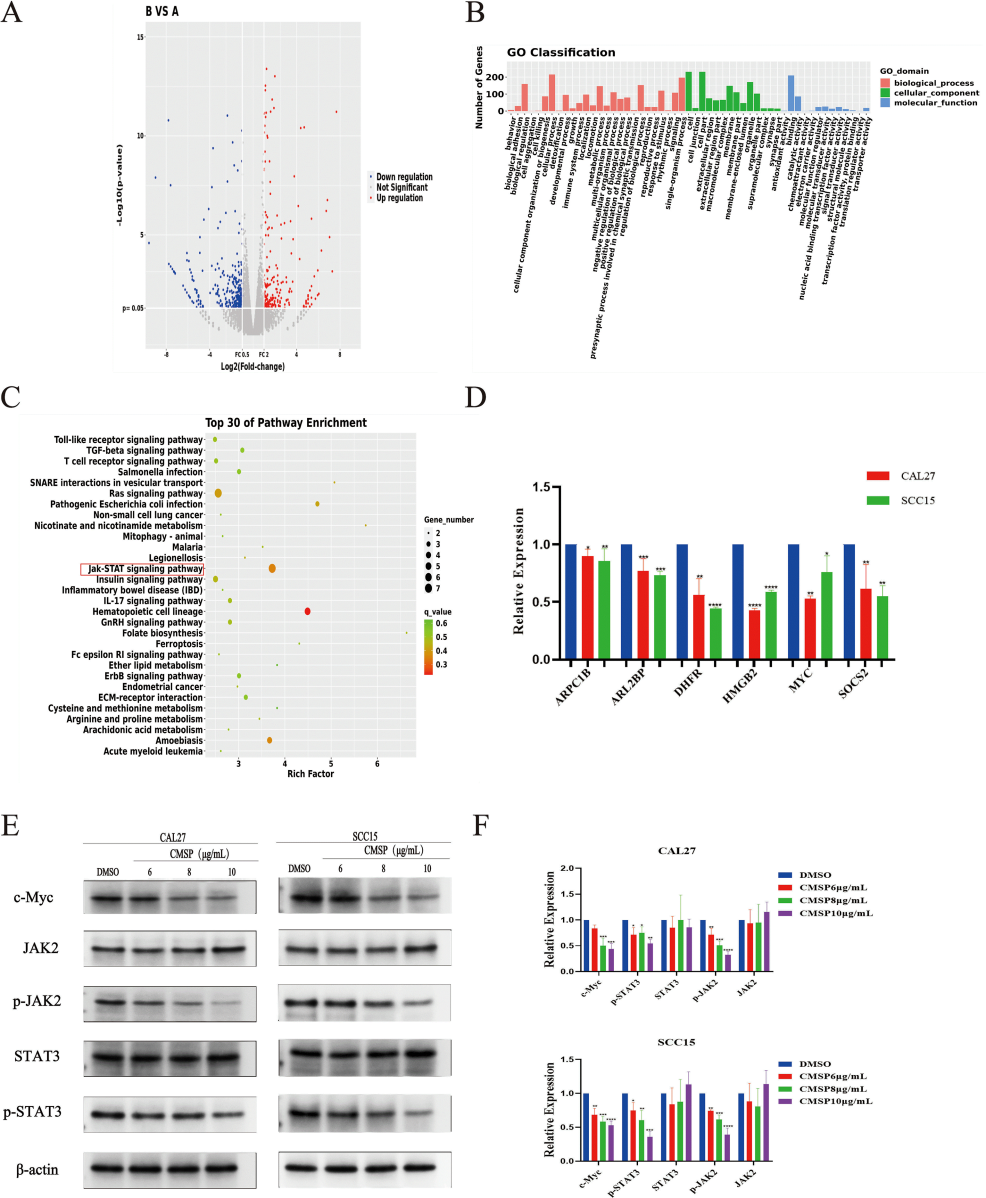
CMSP inhibits cell proliferation through the JAK2/STAT3/c-Myc signal axis. **(A)**Heat map of differentially expressed mRNA. **(B)** Enrichment entries of DEGs GO analysis. **(C)** Enrichment entries of DEGs KEGG pathway analysis(top30). **(D)** qPCR validation of transcriptome sequence data. **(E, F)** Effects of different concentrations of CMSP(6, 8, 10)μg/mL on expression levels of JAK2/STAT3/c-Myc signal axis related proteins in CAL27 and SCC15 cells treated for 48h. β-Actin to control the load. n=3, compared with the control group, *P< 0.05, **P < 0.01, ***P < 0.001, ****P < 0.0001.

To elucidate the molecular mechanisms underlying the anti-proliferative effects of CMSP, we conducted transcriptomic sequencing of CAL27 cells, which exhibited the highest sensitivity to CMSP treatment. Analysis revealed 590 significantly differentially expressed mRNAs, including 258 upregulated and 332 downregulated genes following exposure to 8 μg/mL CMSP (**Figure 5A**). KEGG and GO enrichment analyses performed using R software demonstrated that many of these differentially expressed genes were significantly enriched in the JAK-STAT signaling pathway (**Figures 5B, C**).

From the set of downregulated genes, we selected six candidates—SOCS2, MYC, DHFR, ARPC1B, ARL2BP, PCDHGC3, and HMGB2—based on their biological relevance and previous literature reports, with SOCS2 and MYC being directly associated with JAK-STAT signaling regulation (21–28). RT-qPCR validation confirmed that the mRNA expression levels of these genes were consistent with the transcriptomic findings (**Figure 5D**).

Given the enrichment of CMSP-affected genes in the JAK-STAT pathway, we investigated whether CMSP modulates this pathway to exert its anti-proliferative effects. c-Myc, a key transcriptional target of the JAK/STAT axis, plays a crucial role in promoting cell proliferation. Western blot analysis revealed that CMSP treatment resulted in a dose-dependent downregulation of c-Myc protein levels in both CAL27 and SCC15 cells. Additionally, phosphorylation levels of JAK2 and STAT3—essential activators in this pathway—were significantly suppressed by CMSP, while total JAK2 and STAT3 protein levels remained unchanged (**Figures 5E, F**).

These findings suggest that CMSP suppresses OSCC cell proliferation by inhibiting the JAK2/STAT3/c-Myc signaling axis, highlighting this pathway as a critical target in its anti-tumor activity.

### 3.6 Relationship between MYC expression and OSCC pathogenesis and CMSP response

**Figure 6:**
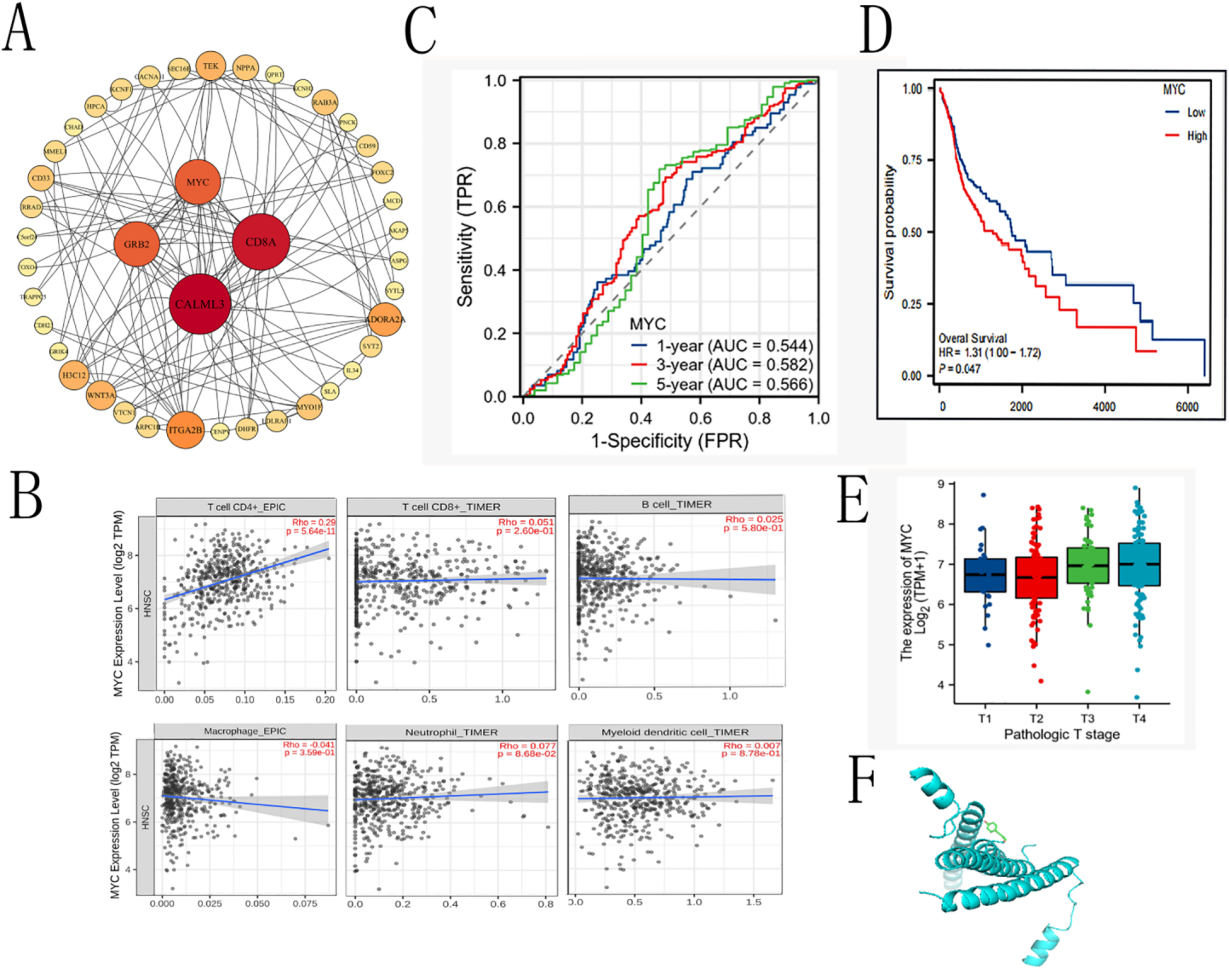
The prognostic value of MYC in OS and DSS of OSCC. **(A)**Cytokine-cytokine receptor interaction. **(B)** Correlation with immune cells. **(C)** Time-dependent ROC curve of MYC. **(D)** The prognostic value of MYC in OS of OSCC. **(E)** expression of MYC in different tumor stages **(F)** dockings with visualization by PYMOL.

To further explore the potential downstream targets through which CMSP modulates OSCC progression, we analyzed the downregulated genes identified via transcriptome sequencing using the STRING online database to construct a PPI network. The resulting network was imported into Cytoscape for topological analysis and visualization (**Figure 6A**). In this network, each edge represents a functional interaction between proteins, with denser connections indicating stronger correlations. Core gene analysis identified CALML3, CD8A, MYC, and EGFR as key nodes with high connectivity.

Given our prior findings implicating CMSP in the regulation of the JAK2/STAT3/c-Myc axis, we further focused on MYC as a potential effector mediating the anti-tumor effects of CMSP. To explore the clinical and immunological relevance of MYC expression in OSCC, we utilized the TIMER2.0 database to assess its correlation with tumor-infiltrating immune cells. The results indicated a statistically significant positive correlation between MYC expression and CD4+ T cell infiltration (P = 5.64 × 10^-^¹¹), while associations with CD8+ T cells, B cells, macrophages, dendritic cells, and neutrophils were not significant (**Figure 6B**). These findings suggest a possible role of MYC in modulating the tumor immune microenvironment, particularly T helper cell responses.

To assess the prognostic value of MYC expression in OSCC, we conducted Kaplan–Meier survival analysis based on publicly available clinical datasets. Patients were stratified into high and low MYC expression groups. Elevated MYC expression was associated with worse overall survival (HR = 1.31, 95% CI: 1.00–1.72, p = 0.047), particularly in advanced stages (stage III–IV) (**Figures 6C–E**). These data support MYC as a negative prognostic biomarker in OSCC.

Finally, to explore the potential interaction between CMSP and MYC, molecular docking simulations were performed using AutoDock Vina. The binding energy between CMSP and the MYC protein was calculated to be −4.2 kcal/mol, indicating a feasible binding affinity under physiological conditions (**Figure 6F**). While these results provide preliminary structural insight into how CMSP might interact with MYC, further biochemical and biophysical validation is required to confirm direct binding.

### 3.7 CMSP inhibits tumor growth in OSCC xenograft models

To validate the *in vivo* anti-tumor efficacy of CMSP, a nude mouse xenograft model was established using CAL27 cells. Mice were treated with either normal saline (control group) or CMSP at a dose of 20 mg/kg every other day. Tumor volumes were measured regularly, and tumor weights were assessed at the end of the 19-day treatment period.

**Figure 7:**
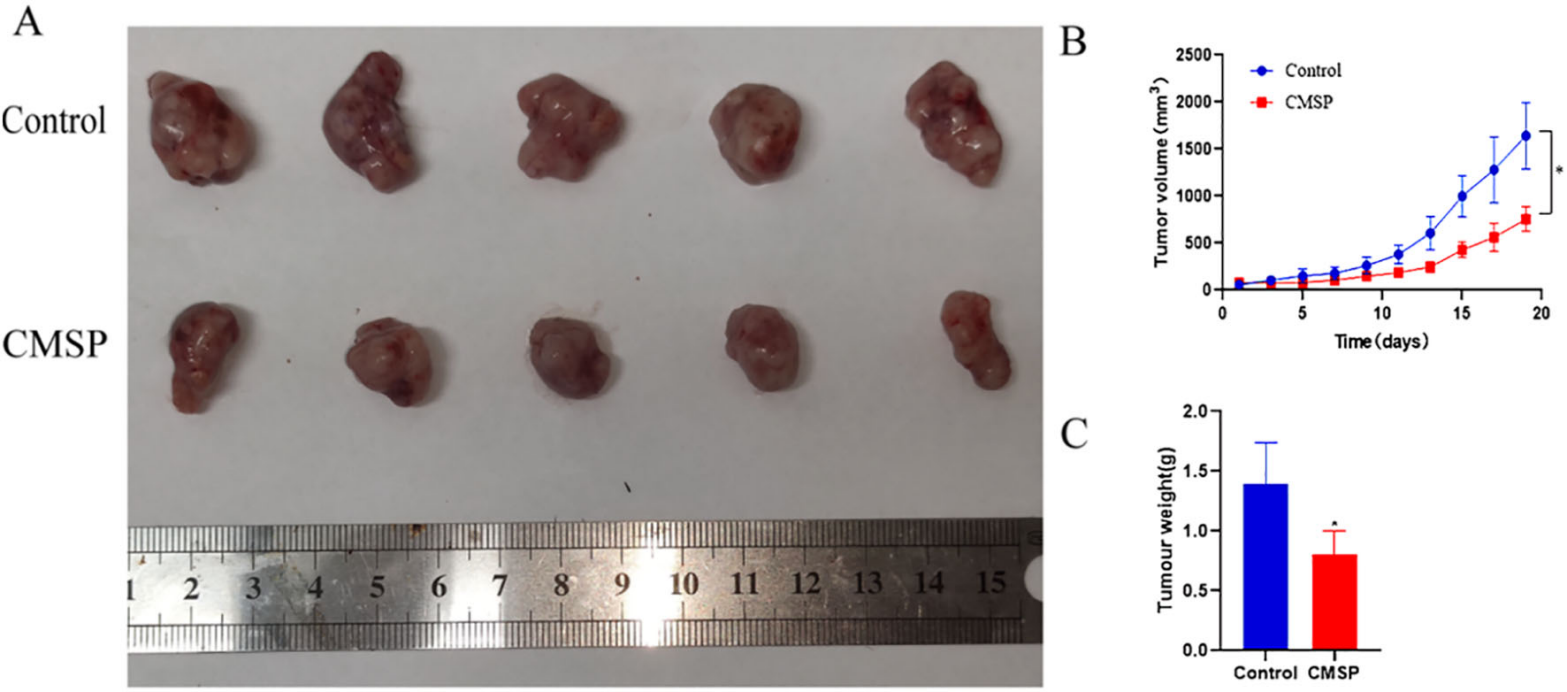
Inhibition of CAL27 cell growth *in vivo* induced by treatment with CMSP. The tumour xenografts sizes were recorded daily in each treatment group. Upon termination of the experiment, the wet tumour weight was measured. Significant inhibition of tumour xenograft growth and weight was recorded in CMSP treated mice. **(A)** Representative images of tumours from different groups are shown. **(B)** tumour growth curves of xenograft tumours. **(C)** Measurement of tumour weights. n=3, compared with the control group, *p< 0. 05.

The results demonstrated that CMSP treatment led to a significant reduction in both tumor growth rate and final tumor volume compared to the control group (**Figures 7A–C**). Specifically, tumor growth curves showed a clear and consistent suppression in the CMSP-treated group throughout the experimental timeline (**Figure 7B**). Additionally, the average tumor weight in CMSP-treated mice was markedly decreased relative to controls (**Figure 7C**). These findings confirm that CMSP exerts substantial *in vivo* anti-tumor activity against OSCC.

## 4 Discussion

This study systematically evaluated the antitumor effects of CMSP on OSCC and explored its underlying mechanisms. *In vitro* experiments demonstrated that CMSP inhibited the proliferation of OSCC cells (CAL27 and SCC15) in a dose- and time-dependent manner (IC_50_: 8.52-13.84 μg/mL; **Figures 1A, B**). CMSP suppressed EMT by upregulating *E-cadherin* and downregulating *Vimentin* (**Figures 2A-C**), induced S phase cell cycle arrest by downregulating *CDK2* and *Cyclin A* (**Figures 4A, B**), and promoted apoptosis by increasing the expression of cleaved *Caspase-3* and *Bax* (**Figures 3A, B**). Transcriptome analysis identified 590 differentially expressed genes, among which the *JAK2/STAT3/c-Myc* pathway was significantly enriched and markedly downregulated following CMSP treatment (**Figures 5A-E**). *In vivo*, administration of CMSP (20 mg/kg) significantly reduced tumor volume and weight in a CAL27 xenograft nude mouse model (**Figures 7A-C**). These findings collectively indicate that CMSP exerts multi-targeted antitumor effects in OSCC, prominently through inhibition of the JAK2/STAT3/c−Myc axis.

CMSP, a natural organic compound, has been shown to exhibit inhibitory effects on various tumors. Previous studies have shown that CMSP exerts antitumor effects in various malignancies, including esophageal squamous cell carcinoma and melanoma, possibly by promoting the phosphorylation of *p38* and *JNK* in the *MAPK* signaling pathway, thereby inducing tumor cell differentiation and apoptosis, or by modulating the tumor immune microenvironment (for example, by promoting macrophage M1 polarization) (7, 10, 15, 16, 29, 30).Consistent with these findings, our study demonstrates that CMSP not only inhibits the proliferation of OSCC cells, but also induces apoptosis and S phase cell cycle arrest by regulating apoptosis-and cell cycle-related proteins such as *caspase-3*, *Bcl-2*, *Bax*, *CDK2*, and *Cyclin A*.

Notably, CMSP treatment led to the downregulation of Cyclin A and CDK2, which are essential for S-phase progression and DNA synthesis. This suggests that CMSP may impair DNA replication dynamics or activate intra-S-phase checkpoints, thereby inducing S-phase arrest. At first glance, the concurrent S-phase arrest and downregulation of Cyclin A observed in CMSP-treated OSCC cells may appear contradictory. However, multiple plausible mechanisms may account for this outcome. First, CMSP may induce replication stress and activate intra-S-phase checkpoints, such as the ATR/Chk1 pathway, which has been reported to suppress Cyclin A expression via proteasomal degradation or transcriptional repression (54, 55). Second, as CMSP also robustly induces apoptosis (**Figure 3**), caspase-mediated cleavage of Cyclin A may contribute to the observed reduction in protein levels (56). Third, CMSP may inhibit upstream transcriptional regulators such as c-Myc, resulting in suppressed Cyclin A transcription. Lastly, the protein expression was assessed 48 hours after treatment, a time point at which Cyclin A may already be depleted due to prior stress or apoptotic signaling. These explanations suggest that the S-phase arrest observed represents a dysfunctional or abortive S phase, rather than classical Cyclin A–driven S-phase progression.

Taken together, these mechanisms suggest that CMSP causes a dysfunctional S-phase arrest rather than a classical Cyclin A driven progression, which is consistent with the behavior of other replication-inhibiting natural compounds (31, 32).

Furthermore, CMSP significantly inhibited the phosphorylation of JAK2 and STAT3 and reduced the expression of c-Myc, a transcription factor known to regulate genes such as CCNA2 (Cyclin A2) and CDK2. Given this regulatory relationship, the suppression of c-Myc provides a plausible explanation for the reduced expression of Cyclin A/CDK2 and the observed S-phase arrest. The JAK2/STAT3/c-Myc axis is known to promote S-phase entry by driving transcription of Cyclin A, E2F targets, and CDKs. Thus, CMSP-induced inhibition of this pathway may disrupt S-phase entry or checkpoint integrity, leading to cell cycle stalling.Although a direct interaction between CMSP and cell cycle regulators cannot be excluded, the current evidence suggests that CMSP induces S-phase arrest primarily through upstream suppression of the JAK2/STAT3/c-Myc pathway.

CMSP was observed to activate both intrinsic and extrinsic apoptotic pathways, suggesting that it may serve as an effective apoptosis inducer to inhibit OSCC progression.These findings are generally consistent with previous studies regarding the anticancer effects of natural organic compounds against oral cancer.Plumbagin, a naphthoquinone compound, has been shown to enhance its anticancer effects by arresting SCC25 cells at the G2/M phase through downregulation of CDK2 and Cyclin B1 expression (31). Similarly, isorhamnetin, a natural flavonoid, can induce G2/M phase arrest in HSC-3 cells by inhibiting Cyclin B1 and CDK2 (31). Research by Pouyfung et al. demonstrated that 8α-tigloyloxyhirsutinolide 13-O-acetate (8αTGH) suppresses the expression of cell cycle regulators CDK1/2 and Cyclin B1, resulting in G2/M arrest in HSC4 cells (32). In contrast, magnolol induces cell cycle arrest at the G0/G1 phase in OC2 and OCSL cells, thereby promoting apoptosis (33). Consistent with these findings, our study shows that CMSP not only inhibits OSCC cell proliferation, but also induces apoptosis and S phase cell cycle arrest by regulating apoptosis- and cell cycle-related proteins such as caspase-3, Bcl-2, Bax, CDK2, and Cyclin A.

The extrinsic (receptor-mediated) and intrinsic (mitochondria-mediated) apoptotic pathways are not mutually independent; rather, they often intersect under various circumstances, with the execution of apoptosis ultimately dependent on the activation of the caspase family of proteins. Previous studies have shown that 7-epitaxol, a major derivative of paclitaxel, can induce depolarization in SCC9 and SCC47 cells, significantly increase the expression of pro-apoptotic Bcl-2 family proteins, key components of the Fas and tumor necrosis factor (TNF) pathways, and enhance the activation of caspase-3, -8, and -9 as well as the cleavage of PARP, thereby promoting apoptosis (34). Similarly, our study found that CMSP induces apoptosis in OSCC cells through the cross-activation of both intrinsic and extrinsic apoptotic pathways. This finding is consistent with previous results and further confirms the critical role of pathway crosstalk in the regulation of apoptosis in OSCC cells.

This study confirmed that CMSP can significantly inhibit the migration and invasion of OSCC cells. The underlying mechanism involves the regulation of proteins closely associated with EMT, such as E-cadherin and Vimentin, thereby blocking the invasive and metastatic potential of OSCC cells. These findings are consistent with previous studies showing that a variety of natural compounds including lycorine, tetrandrine (TET), and baicalin can synergistically inhibit OSCC cell migration and invasion by modulating EMT-related proteins and multiple signaling pathways (35–47). Li et al. reported that resveratrol can effectively inhibit the proliferation and metastasis of OSCC through multi-target mechanisms, including the induction of apoptosis and the inhibition of EMT (48). Kumbhar et al. (2024) demonstrated that andrographolide exerts its antiproliferative effects by inducing apoptosis in tumor cells, which is highly consistent with the findings of this study regarding the anticancer mechanisms of natural products against oral cancer (49).

In recent years, numerous reports have indicated that JAK2 and STAT3 are closely associated with various malignant behaviors in tumor cells, including cell cycle regulation and apoptosis. In OSCC cells, overexpression of miR-141-3p can inhibit cell proliferation, migration, and invasion. The use of a miR-141-3p inhibitor weakens the suppression of the JAK2/STAT3 signaling pathway, suggesting that upregulation of miR-141-3p can inhibit the malignant biological behaviors of OSCC cells through the JAK2/STAT3 signaling pathway (50).

In the present study, CMSP was shown to inhibit JAK2 and STAT3 phosphorylation and downregulate c-Myc expression, which may explain its effects on cell cycle arrest, proliferation, apoptosis, and invasion. Although transcriptomic analysis and molecular docking suggest CMSP targets the JAK2/STAT3/c-Myc axis, direct biochemical evidence of CMSP binding to MYC was not obtained. Nonetheless, the multi-level suppression of this axis by CMSP supports its relevance as a core mechanism contributing to the antitumor activity.

Furthermore, from a pharmacological standpoint, the safety and metabolic characteristics of CMSP support its potential translational application.CMSP is presumed to undergo aldehyde oxidation and Phase II conjugation similar to cinnamaldehyde, which undergoes rapid first-pass metabolism and exhibits limited oral bioavailability (51, 52). *In vivo* studies further showed that the maximum tolerated dose in mice exceeded the effective dose by over 20fold, with only mild hepatic effects at extremely high doses (53). Taken together, the low toxicity profile and reasonable pharmacokinetic features of CMSP highlight its promising potential as a novel anti-tumor candidate derived from traditional Chinese medicine.

Compared to existing inhibitors of the JAK2/STAT3/c-Myc axis—such as STAT3 antisense oligonucleotides or c-Myc inhibitors—CMSP demonstrates a distinct advantage by simultaneously suppressing multiple nodes within the pathway while maintaining a favorable safety profile. This positions CMSP as a potential adjunct or alternative therapeutic strategy for OSCC.

Although CMSP shows promising pharmacokinetic features and low toxicity in preclinical models, comprehensive toxicological studies are needed to evaluate systemic safety, off-target effects, and long-term tolerability. Moreover, while CMSP has demonstrated potential immune-modulatory effects such as M1 macrophage polarization, further investigation is required to understand its role within the broader tumor immune microenvironment, including interactions with T lymphocytes and immune checkpoints. These insights will be crucial for positioning CMSP within the existing OSCC therapeutic landscape and for optimizing its clinical translation.

In conclusion, CMSP exerts antitumor effects in OSCC by suppressing proliferation, inducing apoptosis, arresting the cell cycle in S-phase, and inhibiting EMT. These effects are likely mediated via coordinated inhibition of the JAK2/STAT3/c-Myc pathway. The findings support the potential of CMSP as a promising multi-targeted therapeutic candidate for OSCC.By suppressing this pathway at multiple levels, it may restore sensitivity and complement existing systemic agents. Its favorable profile and potential for topical or nanocarrier delivery suggest CMSP is best suited as an adjunct in resistant or biomarker defined OSCC.

This study is not without certain limitations. First, only two OSCC cell lines (CAL27 and SCC15) were employed in the *in vitro* experiments, which may not fully represent the heterogeneity of OSCC. Second, the relatively small sample size in the xenograft mouse model reduced the statistical power of the *in vivo* analyses. Future studies should therefore incorporate a broader panel of OSCC cell lines and patient derived xenograft models, increase animal numbers to enhance statistical reliability. In addition, comprehensive pharmacokinetic and toxicological studies are warranted to clarify the metabolism and safety profile of CMSP *in vivo*.

## Data Availability

The original contributions presented in the study are included in the article/supplementary material. Further inquiries can be directed to the corresponding author.

## References

[1] Vitório JG Duarte-Andrade FF Dos Santos FPT Fonseca FP Amorim LSD Martins-Chaves RR . Metabolic landscape of oral squamous cell carcinoma. Metabolomics. (2020) 16:105. doi: 10.1007/s11306-020-01727-6, PMID: 33000429

[2] Tan Y Wang Z Xu M Li B Huang Z Qin S . Oral squamous cell carcinomas: state of the field and emerging directions. Int J Oral Sci. (2023) 15:44. doi: 10.1038/s41368-023-00249-w, PMID: 37736748 PMC10517027

[3] Hu X Xiang F Feng Y Gao F Ge S Wang C . Neutrophils promote tumor progression in oral squamous cell carcinoma by regulating EMT and JAK2/STAT3 signaling through Chemerin. Front Oncol. (2022) 12:812044. doi: 10.3389/fonc.2022.812044, PMID: 35155249 PMC8831747

[4] Sacco AG Cohen EE . Current treatment options for recurrent or metastatic head and neck squamous cell carcinoma. J Clin Oncol. (2015) 33:3305–13. doi: 10.1200/JCO.2015.62.0963, PMID: 26351341

[5] Lin SR Chang CH Hsu CF Tsai MJ Cheng H Leong MK . Natural compounds as potential adjuvants to cancer therapy: Preclinical evidence. Br J Pharmacol. (2020) 177:1409–23. doi: 10.1111/bph.14816, PMID: 31368509 PMC7056458

[6] Zhou X Seto SW Chang D Kiat H RazmovskiNaumovski V Chan K . Synergistic effects of Chinese herbal medicine: a comprehensive review of methodology and current research. Front Pharmacol. (2016) 7:201. doi: 10.3389/fphar.2016.00201, PMID: 27462269 PMC4940614

[7] Ma M Zhao LM Yang XX Shan YN Cui WX Chen L . p-Hydroxycinnamaldehyde induces the differentiation of oesophageal carcinoma cells via the cAMP-RhoA-MAPK signalling pathway. Sci Rep. (2016) 6:31315. doi: 10.1038/srep31315, PMID: 27501997 PMC4977536

[8] Yu JS Kim JH Lee S Jung K Kim KH Cho JY . Src/Syk-targeted anti-inflammatory actions of triterpenoidal saponins from Gac (Momordica cochinchinensis) seeds. Am J Chin Med. (2017) 45:459–73. doi: 10.1142/S0192415X17500288, PMID: 28367713

[9] Ai Z Ma C Wan R Yin J Li G Li Y . Anticancer activity and molecular mechanism of momordica cochinchinensis seed extract in chronic myeloid leukemia cells. Nutr Cancer. (2022) 74:3436–46. doi: 10.1080/01635581.2021.2014904, PMID: 34907814

[10] Zhao LM Sun GG Han LN Liu LH Ren FZ Li L . P-Hydroxycinnamaldehyde induces B16-F1 melanoma cell differentiation via the RhoA-MAPK signaling pathway. Cell Physiol Biochem. (2016) 38:2247–60. doi: 10.1159/000445580, PMID: 27188168

[11] Buranrat B Kraiklang R . Momordica cochinchinensis suppresses the human breast cancer cells growth and migration by inhibiting mevalonate pathway. Pharmacogn Magazine. (2023) 19:284–94. doi: 10.1177/09731296231157982

[12] Wang M Zhan Z Xiong Y Zhang Y Li X . Cytotoxic and anti-inflammatory constituents from Momordica cochinchinensis seeds. Fitoterapia. (2019) 139:104360. doi: 10.1016/j.fitote.2019.104360, PMID: 31629869

[13] Shen Y Meng L Sun H Zhu Y Liu H . CochinChina momordica seed suppresses proliferation and metastasis in human lung cancer cells by regulating multiple molecular targets. Am J Chin Med. (2015) 43:149–66. doi: 10.1142/S0192415X1550010X, PMID: 25649746

[14] Zheng L Zhang Y Liu Y Yang XO Zhan Y . Momordica cochinchinensis Spreng. seed extract suppresses breast cancer growth by inducing cell cycle arrest and apoptosis. Mol Med Rep. (2015) 12:6300–10. doi: 10.3892/mmr.2015.4186, PMID: 26252798

[15] Zhao LM Geng YM Sun SP Ren FZ Han LN Shan BE . Differentiation of mouse melanoma B16 cells induced by p-hydroxycinnamaldehyde and its mechanism. Chin J Tumor Biother. (2014) 21:282–7. doi: 10.3872/j.issn.1007-385X.2014.03.008

[16] Wang X Wei S Li W Wei X Zhang C Dai S . P-hydroxycinnamaldehyde induces tumor-associated macrophage polarization toward the M1 type by regulating the proteome and inhibits ESCC *in vivo* and *in vitro*. Int Immunopharmacol. (2023) 119:110213. doi: 10.1016/j.intimp.2023.110213, PMID: 37137266

[17] Xiao L Li X Cao P Fei W Zhou H Tang N . Interleukin-6 mediated inflammasome activation promotes oral squamous cell carcinoma progression via JAK2/STAT3/Sox4/NLRP3 signaling pathway. J Exp Clin Cancer Res. (2022) 41:166. doi: 10.1186/s13046-022-02376-4, PMID: 35513871 PMC9069786

[18] Qureshy Z Li H Zeng Y Rivera J Cheng N Peterson CN . STAT3 activation as a predictive biomarker for ruxolitinib response in head and neck cancer. Clin Cancer Res. (2022) 28:4737–46. doi: 10.1158/1078-0432.CCR-22-0744, PMID: 35929989 PMC10024606

[19] Liu S Qin Z Mao Y Zhang W Wang Y Jia L . Therapeutic targeting of MYC in head and neck squamous cell carcinoma. Oncoimmunology. (2022) 11:2130583. doi: 10.1080/2162402X.2022.2130583, PMID: 36211811 PMC9543056

[20] Duffy MJ O’Grady S Tang M Crown J . MYC as a target for cancer treatment. Cancer Treat Rev. (2021) 94:102154. doi: 10.1016/j.ctrv.2021.102154, PMID: 33524794

[21] Filbey KJ Varyani F Harcus Y Murray J McSorley HJ Maizels RM . Macrophage migration inhibitory factor (MIF) is essential for type 2 effector cell immunity to an intestinal helminth parasite. Front Immunol. (2019) 10:2375. doi: 10.3389/fimmu.2019.02375, PMID: 31708913 PMC6821780

[22] Aggarwal S Bhadana K Singh B Rawat M Mohammad T Al-Keridis LA . Cinnamomum zeylanicum extract and its bioactive component cinnamaldehyde show anti-tumor effects via inhibition of multiple cellular pathways. Front Pharmacol. (2022) 13:918479. doi: 10.3389/fphar.2022.918479, PMID: 35774603 PMC9237655

[23] Fu Y Qiu C Yang Y Lu J Qi Y . CircLPAR3 acts as an oncogene in oral squamous cell carcinoma through regulating the miR-643/HMGB2 network. Biochem Genet. (2022) 60:882–98. doi: 10.1007/s10528-021-10134-y, PMID: 34528144

[24] Peng HY Jiang SS Hsiao JR Hsiao M Hsu YM Wu GH . IL-8 induces miR-424-5p expression and modulates SOCS2/STAT5 signaling pathway in oral squamous cell carcinoma. Mol Oncol. (2016) 10:895909. doi: 10.1016/j.molonc.2016.03.001, PMID: 27038552 PMC5423170

[25] de Guia RM . Stress, glucocorticoid signaling pathway, and metabolic disorders. Diabetes Metab Syndr. (2020) 14:1273–80. doi: 10.1016/j.dsx.2020.06.038, PMID: 32755820

[26] Dang CV . MYC on the path to cancer. Cell. (2012) 149:22–35. doi: 10.1016/j.cell.2012.03.003, PMID: 22464321 PMC3345192

[27] Yasukawa H Sasaki A Yoshimura A . Negative regulation of cytokine signaling pathways. Annu Rev Immunol. (2000) 18:143–64. doi: 10.1146/annurev.immunol.18.1.143, PMID: 10837055

[28] Delmore JE Issa GC Lemieux ME Rahl PB Shi J Jacobs HM . BET bromodomain inhibition as a therapeutic strategy to target c-Myc. Cell. (2011) 146:904–17. doi: 10.1016/j.cell.2011.08.017, PMID: 21889194 PMC3187920

[29] Zhao L Ma M Wu H Zhang C Dai S Dong P . p-Hydroxylcinnamaldehyde slows the progression of 4NQO-induced oesophageal tumourigenesis via the RhoA-MAPK signaling pathway. Mol Carcinog. (2018) 57:1319–31. doi: 10.1002/mc.22847, PMID: 29873419

[30] Ma M Zhang C Xiang XH Deng XQ Dai SL Wei SS . p-Hydroxylcinnamaldehyde from cochinChinamomordica seed reverses resistance to TRAIL in human oesophageal squamous cell carcinoma via the activation of the p38 mitogen-activated protein kinase signalling pathway. Biomed Pharmacother. (2020) 121:109611. doi: 10.1016/j.biopha.2019.109611, PMID: 31731196

[31] Pan ST Ye FF Huang G Qiu JX . Plumbagin enhances the anticancer effects of PF chemotherapy via downregulation of the PI3K/AKT/mTOR/p70S6K pathway in human tongue squamous cell carcinoma. J Oncol. (2023) 2023:8306514. doi: 10.1155/2023/8306514, PMID: 36814557 PMC9940972

[32] Pouyfung P Choonate S Wongnoppavich A Rongnoparut P Rongnoparut K . Anti-proliferative effect of 8α-tigloyloxyhirsutinolide-13-O-acetate (8αTGH) isolated from Vernonia cinerea on oral squamous cell carcinoma through inhibition of STAT3 and STAT2 phosphorylation. Phytomedicine. (2019) 52:238–46. doi: 10.1016/j.phymed.2018.09.211, PMID: 30599904

[33] Huang KJ Kuo CH Chen SH Li CY Lee YR . Honokiol inhibits *in vitro* and *in vivo* growth of oral squamous cell carcinoma through induction of apoptosis, cell cycle arrest and autophagy. J Cell Mol Med. (2018) 22:1894–908. doi: 10.1111/jcmm.13474, PMID: 29363886 PMC5824386

[34] Kumar VB Hsieh MJ Mahalakshmi B Chuang YC Lin CC Lo YS . 7-epitaxol induces apoptosis and autophagy in head and neck squamous cell carcinoma through inhibition of the ERK pathway. Cells. (2021) 10:2633. doi: 10.3390/cells10102633, PMID: 34685613 PMC8534141

[35] Li HS Zhou D Han NN Yan M Ruan M . Experimental study on the inhibition of proliferation and invasion of oral squamous cell carcinoma cells by lycorine via degradation of SCAP protein. Chin J Oral Maxillofac Surg. (2024) 22:29–35. doi: 10.19438/j.cjoms.2024.01.005

[36] Peng KP Tian GX Peng SW Bin DH Zuo QJ Tan Y . Mechanistic study on tetrandrine inhibiting the invasive potential of oral squamous cell carcinoma via modulation of the HIF-1α/PD-1/PD-L1 axis. J Hunan Univ Chin Med. (2023) 43:1971–7. doi: 10.3969/j.issn.1674-070X.2023.11.006

[37] Pang ZZ Cai ZY Wang L . Cordycepin inhibits the proliferation, migration, and invasion of oral squamous cell carcinoma HSC3 cells by regulating miR-524-5p expression. China Pharm. (2021) 24:482–8. doi: 10.3969/j.issn.1008-049X.2021.03.015

[38] Chen H Wang C Qi M Ge L Tian Z Li J . Anti-tumor effect of rhaponticum uniflorum ethyl acetate extract by regulation of peroxiredoxin1 and epithelial-to-mesenchymal transition in oral cancer. Front Pharmacol. (2017) 8:870. doi: 10.3389/fphar.2017.00870, PMID: 29218012 PMC5703707

[39] Wang C Ma XL Cheng RQ Zhang JJ Yang L . Effect of naringenin-mediated miR-182-5p regulation on migration and IL-6/IL-8 secretion of oral squamous cell carcinoma BcaCD885 cells. Chin J Immunol. (2021) 37:463–8. doi: 10.3969/j.issn.1000-484X.2021.04.016

[40] Wang GT Huang S . Effects and mechanism of baicalein on the proliferation and invasion of human oral squamous cell carcinoma cells. Chin J Appl Physiol. (2018) 34:536–40. 10.12047/j.cjap.5682.2018.119 31032589

[41] Zhong WD Liu RJ Guan HB Chen GS . Inhibitory effect of baicalin on human tongue squamous cell carcinoma SCC15 cells. Prev Treat Stomatol Dis. (2019) 27:226–30. doi: 10.12016/j.issn.2096-1456.2019.04.004

[42] Guo JJ Wang C Zhang WJ Du C Li GL . Effects of combined treatment with baicalein and baicalin on the proliferation and migration of oral squamous cell carcinoma cells. Stomatology. (2020) 40:393–8. doi: 10.13591/j.cnki.kqyx.2020.05.002

[43] Huang CC Li QL Li DQ Huang P Qin X . *In vivo* experimental study on the inhibitory effect of berberine on the proliferation of oral squamous cell carcinoma. J Clin Stomatol. (2019) 35:387–90. doi: 10.3969/j.issn.1003-1634.2019.07.001

[44] Sun XG Yang WC Liu YJ Yang X . Effects of theaflavins on the biological behavior of oral squamous cell carcinoma via regulation of the Snail/Slug signaling pathway. Tianjin Med J. (2023) 51:1164–70. doi: 10.11958/20230202

[45] Yang WE Ho YC Tang CM Hsieh YS Chen PN Lai CT . Duchesnea indica extract attenuates oral cancer cells metastatic potential through the inhibition of the matrix metalloproteinase-2 activity by down-regulating the MEK/ERK pathway. Phytomedicine. (2019) 63:152960. doi: 10.1016/j.phymed.2019.152960, PMID: 31280137

[46] Adnan M Jairajpuri DS Chaddha M Khan MS Yadav DK Mohammad T . Discovering tuberosin and villosol as potent and selective inhibitors of AKT1 for therapeutic targeting of oral squamous cell carcinoma. J Pers Med. (2022) 12:1083. doi: 10.3390/jpm12071083, PMID: 35887580 PMC9322152

[47] Dai Q Cui N Liu WZ . Effect of baicalin on proliferation, apoptosis, and invasion of oral squamous cell carcinoma cells through regulation of the JAK2/STAT3 signaling pathway. Shaanxi Med J. (2024) 53:179–83.

[48] Li B Allela OQB Alkhazali WH Vadia N Jyothi R Panigrahi R . Resveratrol in oral cancer: a systematic review of preclinical studies on its anticancer mechanisms and therapeutic potential. Med Oncol. (2025) 42:329. doi: 10.1007/s12032-025-02903-1, PMID: 40653555

[49] Kumbhar GM Jadhav AD Kheur S Vaibhav Sunil L . Andrographolide demonstrates anti-proliferative activity in oral cancer by promoting apoptosis, the programmed cell death process. Iran J Basic Med Sci. (2024) 27:1300–8. doi: 10.22038/ijbms.2024.76691.16599, PMID: 39229580 PMC11366947

[50] Cao M Tian K Sun W Xu J Tang Y Wu S . MicroRNA-141-3p inhibits the progression of oral squamous cell carcinoma via targeting PBX1 through the JAK2/STAT3 pathway. Exp Ther Med. (2022) 23:97. doi: 10.3892/etm.2021.11020, PMID: 34976139 PMC8674974

[51] Han R Liu H Zhang Y Lv G . Cinnamaldehyde: Pharmacokinetics, anticancer properties and therapeutic potential (Review). Mol Med Rep. (2024) 30:163. doi: 10.3892/mmr.2024.13287, PMID: 38994757 PMC11267250

[52] Kim SO Kim MR Kim HJ Yoon JH Lee H Ahn SG . 2′-Hydroxycinnamaldehyde shows antitumor activity against human oral squamous cell carcinoma cell line SCC-15. Anticancer Res. (2010) 30:489–94. 20332459

[53] Ma M Bai HY Li XY Wang YH Zhao RY Dai SL . Toxicological effect of p-hydroxylcinnamaldehyde extract from the CochinChina momordica seeds on mice. Carcinogene Teratogene Mutagene. (2018) 30:452–456,462. doi: 10.3969/j.issn.1004-616x.2018.06.007

[54] He K He J Wang S Yan J . Regulation of cell cycle and apoptosis by silibinin in human hepatoma cell lines. J Genet Genomics. (2010) 37:249–55. doi: 10.1016/S1673-8527(09)60043-6, PMID: 20439101

[55] Otto T Sicinski P . Cell cycle proteins as promising targets in cancer therapy. Nat Rev Cancer. (2017) 17:93–115. doi: 10.1038/nrc.2016.138, PMID: 28127048 PMC5345933

[56] Sozmen M Tunca R Dag Erginsoy S . Cyclin A expression is associated with apoptosis and mitosis in murine fibrosarcomas. Exp Toxicol Pathol. (2009) 61:41–9. doi: 10.1016/j.etp.2008.05.009, PMID: 18621517

